# Evaluation of prognostic risk factors of triple-negative breast cancer with ^18^F-FDG PET/CT parameters, clinical pathological features and biochemical indicators

**DOI:** 10.3389/fcell.2024.1421981

**Published:** 2024-09-04

**Authors:** Lei Zhu, Xin Yang, Jiying Zhang, Shuling Wang, Yulong Wang, Xing Wan, Xiang Zhu, Xiuyu Song, Zhongsheng Tong, Meng Yang, Weipeng Zhao

**Affiliations:** ^1^ Department of Molecular Imaging and Nuclear Medicine, Tianjin Medical University Cancer Institute and Hospital, National Clinical Research Center for Cancer, Tianjin’s Clinical Research Center for Cancer, Key Laboratory of Cancer Prevention and Therapy, Tianjin, China; ^2^ Department of Breast Oncology, Key Laboratory of Breast Cancer Prevention and Therapy, National Clinical Research Center for Cancer, Tianjin Medical University Cancer Institute and Hospital, Tianjin, China; ^3^ Tianjin Cancer Institute, National Clinical Research Center for Cancer, Key Laboratory of Molecular Cancer Epidemiology of Tianjin, Tianjin’s Clinical Research Center for Cancer, Key Laboratory of Cancer Prevention and Therapy, Tianjin Medical University Cancer Institute and Hospital, Tianjin Medical University, Tianjin, China

**Keywords:** TNBC, ^18^F-FDG PET/CT, clinical pathological features, biochemical indicators, prognosis

## Abstract

**Introduction:**

Breast cancer is a heterogeneous disease comprising various molecular subtypes, including Luminal A, Luminal B, human epidermal growth factor receptor-2 (HER2) positive, and triple negative types, each with distinct biological characteristics and behaviors. Triple negative breast cancer (TNBC) remains a particularly challenging subtype worldwide. Our study aims to evaluate whether Fluorodeoxyglucose Positron Emission Tomography/Computed Tomography (^18^F-FDG PET/CT) parameters, clinical pathological features, and biochemical indicators serve as prognostic risk factors for TNBC. Additionally, we explore correlations between biochemical indicators and ^18^F-FDG PET/CT parameters.

**Methods:**

We conducted a retrospective analysis of 95 TNBC patients who underwent preoperative ^18^F-FDG PET/CT examinations at Tianjin Medical University Cancer Institute and Hospital from 2013 to 2018. Collected data included ^18^F-FDG PET/CT parameters, clinical and pathological features, and biochemical indicators. We used Kaplan-Meier survival analysis and multivariate Cox regression analysis to evaluate associations between ^18^F-FDG PET/CT parameters/biochemical indicators and disease free survival (DFS)/overall survival (OS). The log-rank test determined significant differences in survival curves, and the Spearman correlation coefficient analyzed correlations between quantitative variables. Visualization and analysis were performed using R packages.

**Results:**

Among 95 TNBC patients, mean standardized uptake value (SUV_mean_) was significantly correlated with DFS. Fasting blood glucose (FBG), α- L-fucosylase (AFU) and Creatine kinase (CK) were independent predictors of DFS, while Precursor albumin (PALB) and CK were independent predictors of OS. FBG showed correlations with SUV_peak_ and SUV_mean_, and CK was correlated with peak standardized uptake value (SUV_peak_). Our results indicated that ^18^F-FDG PET/CT parameters and biochemical indicators may constitute a new prognostic model for TNBC patients post-surgery.

**Discussion:**

We found that SUV_mean_, FBG, AFU and CK are predictive factors for DFS in TNBC patients post-surgery, while PALB and CK are predictive factors for OS, which prompts us to pay more attention to these indicators in clinical practice. Also ^18^F-FDG PET/CT parameters and biochemical indicators have potential utility in constituting a new prognostic model for TNBC patients post-surgery.

## 1 Introduction

According to Sung H. et al. 2021, female breast cancer (BC) has surpassed lung cancer as the most commonly diagnosed cancer globally, posing a significant health challenge (Sung et al., 2021). BC is a heterogeneous disease with diverse molecular subtypes (Yan et al., 2024). Triple negative breast cancer (TNBC) is characterized by the lack of human epidermal growth factor receptor-2 (HER2), estrogen receptor and progesterone receptor expression (Wolff et al., 2013), accounting for 10%–20% of all BC patients (Alam et al., 2022). It is the most aggressive subtype with poor prognosis and high risk of mortality (Bergin and Loi, 2019).

Imaging plays a pivotal role in the screening, diagnosis, staging, restaging, and treatment planning of BC (Paydary et al., 2019). Although Positron Emission Tomography/Computed Tomography (PET/CT) is not routinely used for early cancer staging, it is crucial for staging high-risk patients (Cardoso et al., 2019). Fluorodeoxyglucose (^18^F-FDG), a glucose analogue that is taken up by cells with high glucose utilization rates in the body. Before its radioactive decay, its metabolic breakdown or utilization is inhibited due to fluoride at the 2’position in the molecule. Therefore, the distribution of ^18^F-FDG can well reflect the distribution of glucose uptake and phosphorylation by cells in the body (Fowler and Ido, 2002). Thus, ^18^F-FDG is the most commonly used radiopharmaceutical in oncology and can serve as a metabolic biomarker for evaluating tumor glycolytic activity (Boellaard et al., 2015).

TNBC typically exhibits high ^18^F-FDG uptake. Studies have shown that TNBC presents the highest baseline standardized uptake value (SUV), making it a suitable research subject. Humbert et al. 2012’s study found that TNBC presented the highest baseline SUV (Humbert et al., 2012). And de Mooij C. M. et al. 2023 confirmed that Luminal tumours had the lowest and TNBC tumors had the highest max Standardized Uptake Value (SUV_max_) (de Mooij et al., 2023). Additionally, Koo H. R. et al. 2014 also found that triple-negative and HER2-positive breast cancers showed higher SUV_max_ values than luminal A tumours (Koo et al., 2014). Therefore, we selected TNBC patients as the research subjects. In our study, we first evaluated the predictive role of ^18^F-FDG PET/CT parameters, clinical pathological features, and biochemical indicators on the prognosis of TNBC, and explored correlations between biochemical indicators and ^18^F-FDG PET/CT parameters.

## 2 Materials and methods

### 2.1 Patients

Our study included 95 adult TNBC patients who underwent a preoperative ^18^F-FDG PET/CT examination at Tianjin Medical University Cancer Institute and Hospital from 2013 to 2018. Inclusion criteria: 1) Adult female patients; 2) TNBC patients confirmed by pathology; 3) Patients underwent surgical resection treatment; 4) Patients underwent a preoperative ^18^F-FDG PET/CT examination; 5) Patients with relatively complete clinical and pathological features, biochemical indicators, and other case data; 6) Patients with relatively complete follow-up data.

### 2.2 ^18^F-FDG PET/CT imaging acquisition and analysis

All ^18^F-FDG PET/CT scans were performed with a 64 multislice-detector PET/CT scanner (Discovery MI PET/CT; General Electric Healthcare, Waukesha, WI, United States). Patients were fasted 4–6 h before the examination. The blood glucose levels were determined and controlled at <140 mg/dL before the administration of ^18^F-FDG (3.7 MBq per kg body weight). The protocol included an initial CT scan (120 kV, 100 mA, and a slice thickness of 5 mm). PET images from the head to the mid-thigh were acquired in 3-dimensional mode without breath-holding, with an acquisition time of 2 min per bed position (for a total of 6–8 bed positions) after CT scanning. The CT-based, attenuation-corrected PET images were reconstructed with an iterative algorithm. The attenuation-corrected PET, CT, and fused PET/CT results were reviewed and analyzed by 2 nuclear medicine physicians with 5 and 10 years of diagnostic experience in diagnosis. The mass in the breast was selected as the region of interest. The volume-based parameters, including SUV_max_, peak standardized uptake value (SUV_peak_), mean standardized uptake value (SUV_mean_), Metabolic Tumor Volume (MTV), Total Lesion Glycolysis (TLG) and Total Metabolic Tumor Volume (TMTV), were obtained with PET VCAR, the semi-quantitative software of the GE workstation. The estimated threshold was 2.5. TLG was automatically calculated with MTV multiples of the SUV_mean_.

^18^F-FDG PET/CT parameters: 1) SUV is a commonly used semi quantitative indicator in PET/CT tumor diagnosis, reflecting the highest local FDG metabolic activity of the lesion, including SUV_max_, SUV_mean_, and SUV_peak_. In the past, SUV_max_ = 2.5 is commonly used as the diagnostic threshold, and lesions with SUV_max_>2.5 are considered malignant, lesions with SUV_max_<2.0 are considered benign, and the critical range of SUV_max_ is between 2.0 and 2.5 (Nguyen et al., 2015). At present, changes in SUV_max_ are often used in clinical practice as an important indicator to determine therapeutic efficacy, and to indicate the degree of malignancy of tumors (Xu and Wang, 2020). 2) MTV is a volume parameter and is considered as the volume of all voxels included in the region of interest (Bouron et al., 2022). 3) TMTV is calculated as the sum of the metabolic tumor volume of all lesions, reflecting the tumor burden along with a metabolically active lesion within the breast ± regional lymph node (s) (Najid et al., 2023). 4) TLG is equal to MTV times SUV_mean_ in each lesion (Schöder and Moskowitz, 2016), and is a comprehensive parameter representing tumor metabolic activity and metabolic volume, which helps to clinically understand the glucose load at the site of the lesion.

### 2.3 Data collection

Clinical and pathological features, as well as biochemical indicators of 95 TNBC patients were collected by reviewing the patient’s medical charts.

Age, diabetes mellitus, menopause, maximum longitudinal diameter of tumor, maximum transverse diameter of tumor, clinical stage, chemotherapy response, histological grading, axillary lymph node metastasis, Ki67, P53, EGFR, and CK5/6 at diagnosis were obtained in clinical and pathological features.

N terminal pro B type natriuretic peptide, Alanine aminotransferase, Aspartate aminotransferase, Precursor albumin (PALB), Albumin (ALB), Albumin percentage, α1 globulin percentage, α2 globulin percentage, β1 globulin percentage, β2 globulin percentage, γ globulin percentage, Total bilirubin, Direct bilirubin, Alkaline phosphatase, γ-Glutamyl transferase, Cholinesterase, Lactate dehydrogenase, α- L-fucosylase (AFU), Glycylprolyl dipeptidyl aminopeptidase, Total cholesterol, Triglyceride, High density lipoprotein cholesterol, Low density lipoprotein cholesterol, Fasting blood glucose (FBG), Flavin mononucleotide, Glycosylated hemoglobin, Serum creatinine, Serum uric acid, Blood urea nitrogen, β2-Microglobulin (β2MG), Creatine kinase (CK), and creatine kinase isoenzyme were obtained in Biochemical indicators. Biochemical indicators detection methods include two-point endpoint method, continuous monitoring method, etc.

Disease free survival (DFS) is defined as the time from surgical resection to local recurrence, metastasis, or follow-up deadline, while overall survival (OS) is defined as the time from surgical resection to death due to any reason or follow-up deadline. And survival results were collected by following up patients or their families by phone.

### 2.4 Data analysis

Kaplan-Meier survival and multivariate Cox regression analysis implemented in the R package were used to analyze associations between ^18^F-FDG PET/CT parameters/biochemical indicators and DFS/OS. The log-rank test was used to determine significant differences of survival curves stratified by median of parameters for quantitative data. Additionally, the Spearman correlation coefficient was used to analyze the correlation between two quantitative variables. We used R package ggcorrplot to visualize a correlation matrix between ^18^F-FDG PET/CT parameters and biochemical indicators exclude NA data and used R RMS package to generate the nomogram and calibration curve. A two-sided *P* < 0.05 was considered statistically significant. Median overall survival time and 95% CIs are reported where relevant. The statistical analysis in this study was generated using R 4.3.1.

## 3 Result

### 3.1 Patient characteristics

A total of 95 patients who met the inclusion criteria were included in this study. Most of the patients were diagnosed at 40–59 years old, with a median age of 51 years old (25–75 years old). Six patients had diabetes (6.32%), and 51 patients had menopause (65.38%), while the clinical stage was mainly IIA-IIIA (77.41%). In addition, 44 patients had axillary lymph node metastasis (51.16%). Moreover, the median DFS follow-up time was 2,533 days (193–3,955 days), and the median OS follow-up time was 2541.5 days (203–3,955 days) (Table 1).

**TABLE 1:** Patient characteristics.

Patient information	Number	%
Age, years		
25–39	15	15.79
40–59	60	63.16
60–75	20	21.05
Diabetes		
Yes	6	6.32
No	89	93.68
Menopause		
Yes	51	65.38
No	27	34.62
Clinical stage		
IA	3	3.23
IIA	18	19.35
IIB	25	26.88
IIIA	29	31.18
IIIB	8	8.60
IIIC	9	9.68
IV	1	1.08
Axillary lymph node metastasis		
Yes	44	51.16
No	42	48.84
DFS follow-up days		
<1 year (194–326 days)	7	9.72
1–5 years (449–1,011 days)	6	8.33
>5 years (1,865–3,955 days)	59	81.95
OS follow-up days		
<1 year (203 days)	1	1.47
1–5 years (408–1,011 days)	7	10.29
>5 years (1,865–3,955 days)	60	88.24

### 3.2 Kaplan Meier survival curves

#### 3.2.1 ^18^F-FDG PET/CT parameters

**FIGURE 1:**
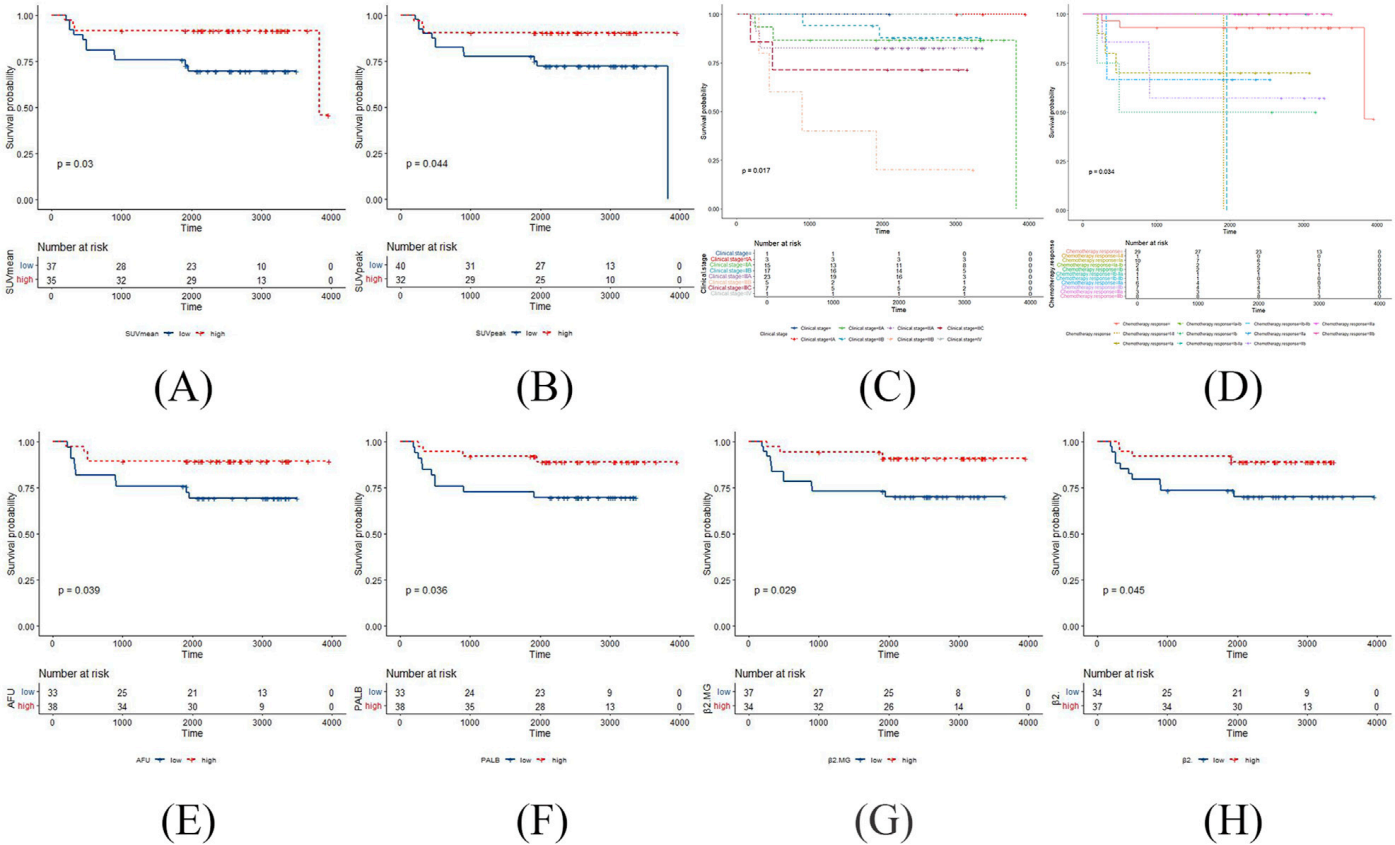
The positive results in the Kaplan Meier survival curves of DFS in TNBC patients post-surgery surgery. **(A)** SUV_mean_ with DFS; **(B)** SUV_peak_ with DFS; **(C)** clinical stage with DFS; **(D)** chemotherapy response with DFS; **(E)** AFU with DFS; **(F)** PALB with DFS; **(G)** β2MG with DFS; **(H)** β2 globulin percentage with DFS.

According to the Kaplan Meier survival curves of DFS, SUV_mean_ and SUV_peak_ had statistically significant log-rank tests in the ^18^F-FDG PET/CT parameters (*p* = 0.03 and *p* = 0.044, respectively) (Figure 1). However, no ^18^F-FDG PET/CT parameters were statistically significant log-rank tests in the Kaplan Meier survival curves of OS. The negative results are shown in Supplementary Figure S1.

#### 3.2.2 Clinical pathological features

**FIGURE 2:**
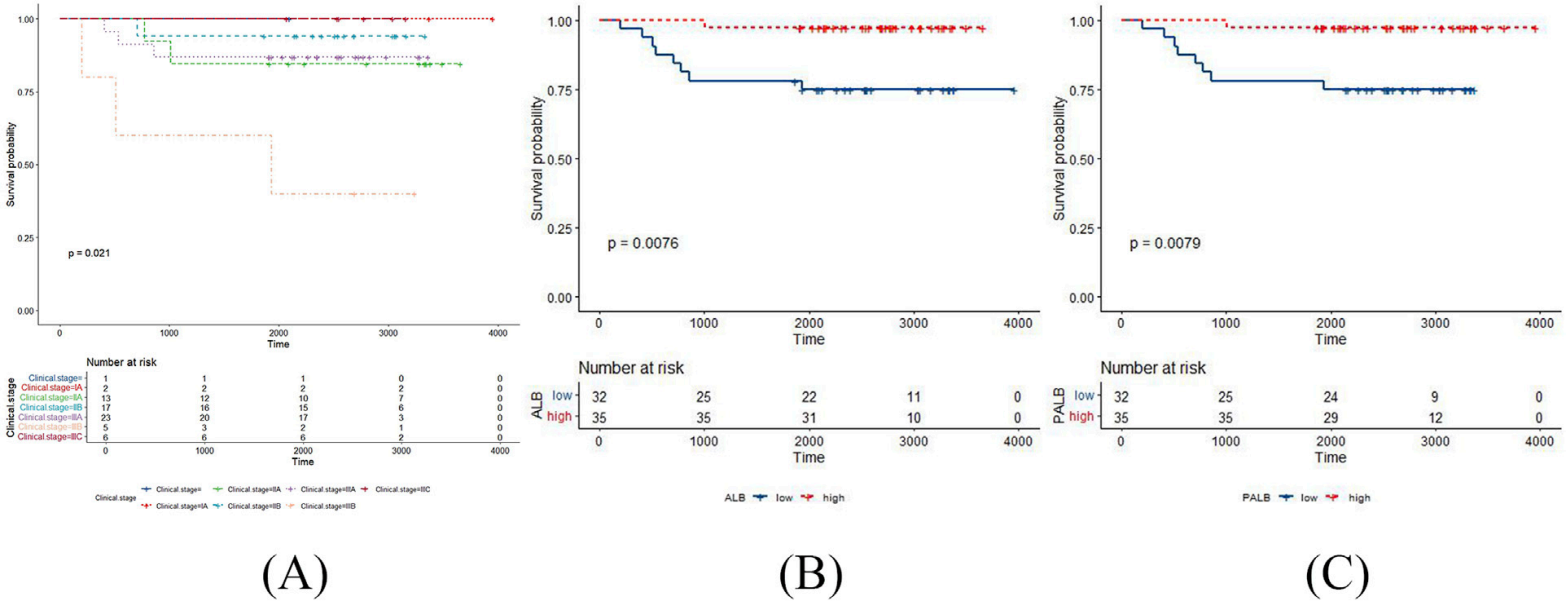
The positive results in the Kaplan Meier survival curves of OS in TNBC patients post-surgery. **(A)** clinical stage with OS; **(B)** ALB with OS; **(C)** PALB with OS.

According to the Kaplan Meier survival curves of DFS, clinical stage and chemotherapy response had statistically significant log-rank tests (*p* = 0.017 and *p* = 0.034, respectively) (Figure 1). Meanwhile, clinical stage had statistically significant log-rank tests in the Kaplan Meier survival curves of OS (*p* = 0.021) (Figure 2). The negative results are shown in Supplementary Figures S2, S3.

#### 3.2.3 Biochemical indicators

According to the Kaplan Meier survival curves of DFS, AFU, PALB, β2MG and β2 globulin percentage had statistically significant log-rank tests (*p* = 0.039, *p* = 0.036, *p* = 0.029, and *p* = 0.045, respectively) (Figure 1). Meanwhile, ALB, and PALB had statistically significant log-rank tests in the Kaplan Meier survival curves of OS (*p* = 0.0076, and *p* = 0.0079, respectively) (Figure 2). The negative results are shown in Supplementary Figures S4, S5.

### 3.3 Cox regression analysis

#### 3.3.1 ^18^F-FDG PET/CT parameters

**FIGURE 3:**
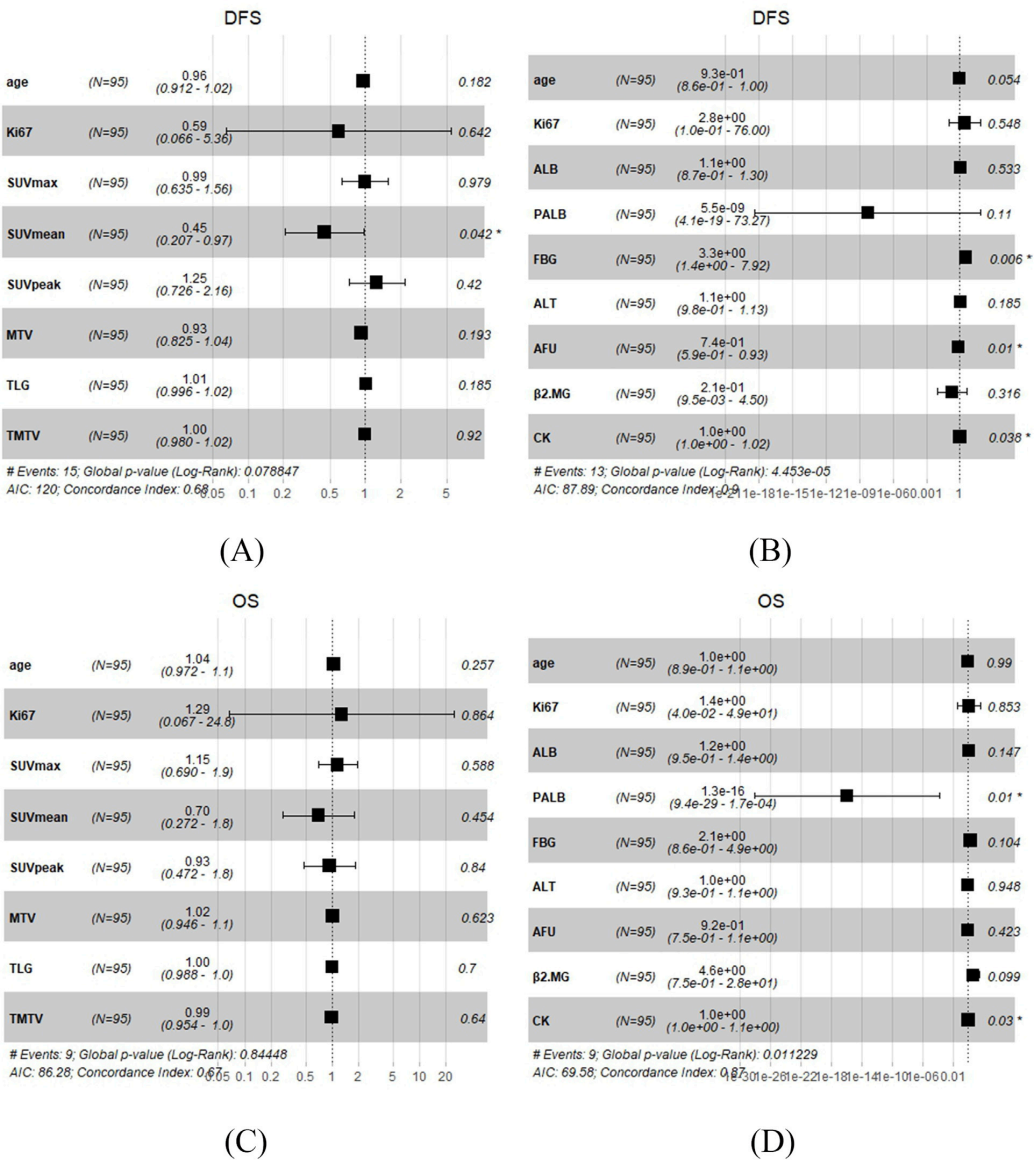
Multivariate Cox regression analysis in TNBC patients undergoing surgery. **(A)** ^18^F-FDG PET/CT parameters with DFS. **(B)** Biochemical indicators with DFS. **(C)** ^18^F-FDG PET/CT parameters with OS. **(D)** Biochemical indicators with OS.

In the univariate analysis, there was no ^18^F-FDG PET/CT parameters were correlated with DFS or OS (Supplementary Table S1). Except ^18^F-FDG PET/CT parameters, we incorporated age and Ki67 at diagnosis for the multivariate analysis, and the result showed that SUV_mean_ was correlated with DFS (*p* = 0.042), nevertheless, there was no ^18^F-FDG PET/CT parameters were correlated with OS (Figure 3). Based on the above results, it can be concluded that SUV_mean_ was significantly correlated with DFS.

#### 3.3.2 Biochemical indicators

In the univariate analysis, the results showed that ALT, PALB, AFU, β2MG, and CK were significantly correlated with DFS (*p* = 0.017, *p* = 0.011, *p* = 0.003, *p* = 0.038, and *p* = 0.026, respectively), while PALB, ALB, and FBG were significantly correlated with OS (*p* = 0.005, *p* = 0.045, and *p* = 0.008, respectively) (Table 2). And the whole results are shown in Supplementary Tables S2, S3. Based on univariate COX analysis result, we choose ALT, PALB, AFU, β2MG, CK, ALB, and FBG and incorporate age and Ki67 at diagnosis for the multivariate analysis. The results showed that FBG, AFU and CK were significantly correlated with DFS (*p* = 0.006, *p* = 0.01, and *p* = 0.038, respectively), meanwhile PALB and CK were significantly correlated with OS (*p* = 0.01 and *p* = 0.03, respectively) (Figure 3). Based on the above results, it can be concluded that FBG, AFU and CK were independent predictors of DFS, while PALB and CK were independent predictors of OS.

**TABLE 2:** Univariate Cox regression analysis of biochemical indicators with DFS and OS in TNBC patients post-surgery.

Biochemical indicators	DFS		OS	
	HR (95% CI)	*P* Value	HR (95% CI)	*P* Value
ALT	1.06 (61.011–1.112)	0.017		
PALB	0 (0–000.099)	0.011	0 (0–0.024)	0.005
AFU	0.848 (0.761–0.946)	0.003		
β2MG	0.178 (0.035–0.908)	0.038		
CK	1.011 (1.003–1.019)	0.005		
ALB			0.919 (0.846–0.998)	0.045
FBG			2.049 (1.21–3.468)	0.008

### 3.4 The correlation between ^18^F-FDG PET/CT parameters and biochemical indicators

**FIGURE 4:**
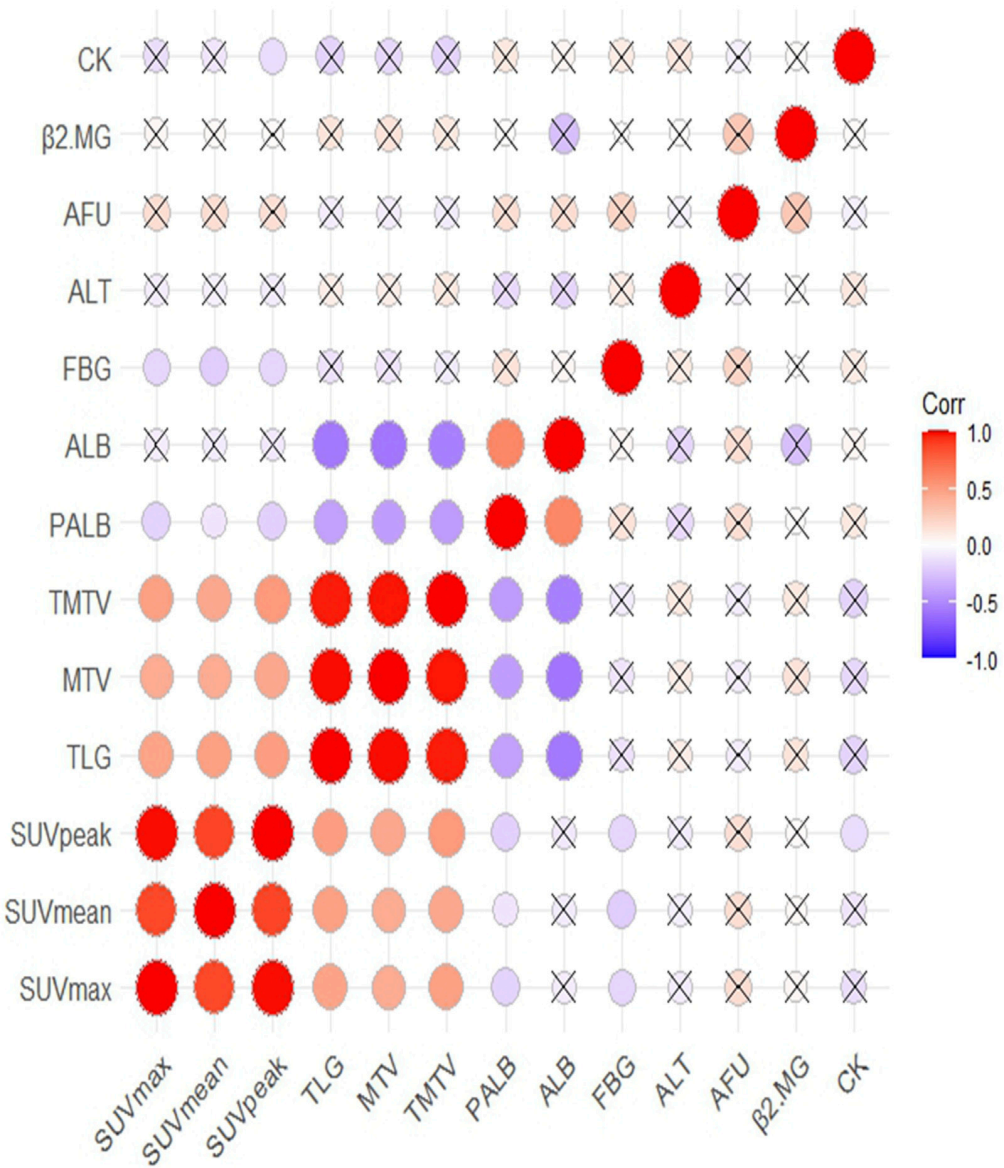
The correlation between ^18^F-FDG PET/CT parameters and biochemical indicators and correlation significance level presented as without cross.

The Spearman correlation coefficient was used to analyze the correlation between ^18^F-FDG PET/CT parameters and ALT, PALB, AFU, β2MG, CK, ALB, and FBG. The results showed that PALB was correlated with all ^18^F-FDG PET/CT parameters; ALB was correlated with TLG, MTV, and TMTV; FBG was correlated with SUV_max_, SUV_mean_, and SUV_peak_; and CK was correlated with SUV_peak_ (Supplementary Table S4; Figure 4). Therefore, ^18^F-FDG PET/CT parameters and biochemical indicators may constitute a new prognostic model for TNBC patients post-surgery.

### 3.5 Construction and validation of the nomogram

**FIGURE 5:**
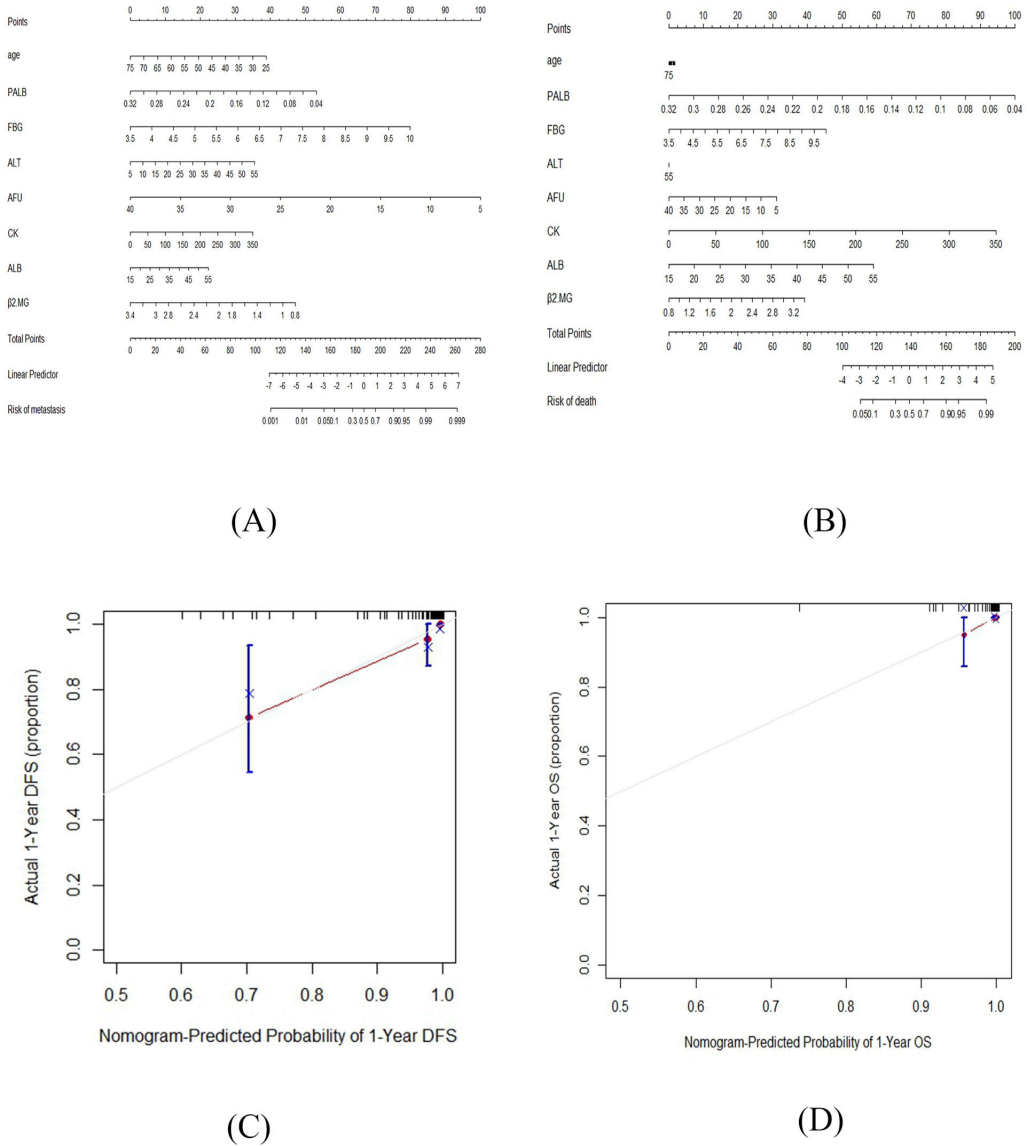
The nomograms for the prediction of 1-year DFS and 1-year OS of TNBC patients post-surgery and the calibration curves of the prognostic nomograms. **(A)** The nomogram for the prediction of 1-year DFS. **(B)** The nomogram for the prediction of 1-year OS. **(C)** The calibration curve of the prognostic nomogram of 1-year DFS. **(D)** The calibration curve of the prognostic nomogram of 1-year OS.

**FIGURE 6:**
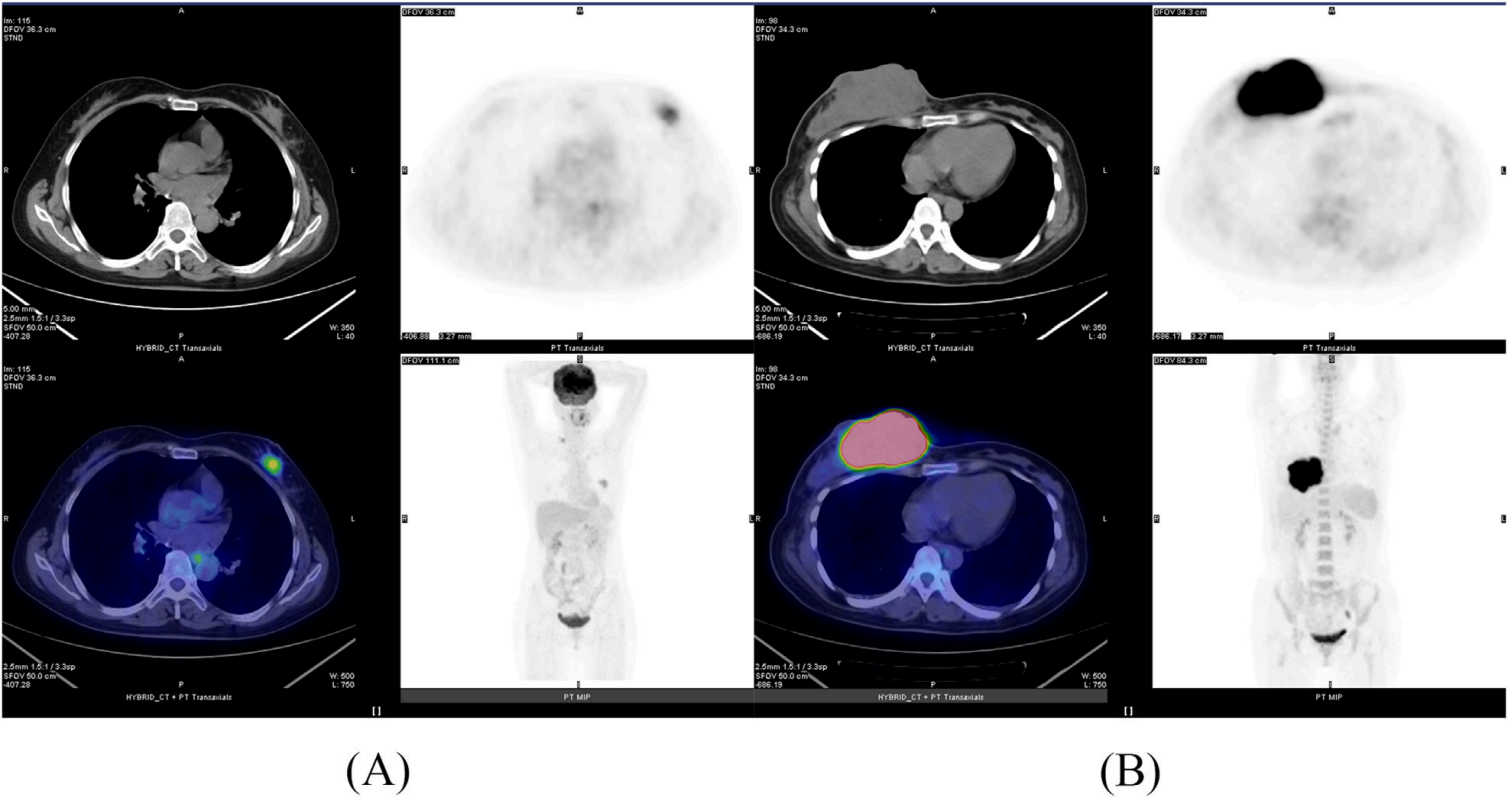
The ^18^F-FDG PET/CT images of two patients. **(A)** Patient A: SUV_max_ = 4.60, SUV_mean_ = 3.28, SUV_peak_ = 3.42. **(B)** Patient B: SUV_max_ = 28.55, SUV_mean_ = 10.99, SUV_peak_ = 24.22.

Based on the above results, two nomograms were developed for the prediction of 1-year DFS and 1-year OS of these patients (Figure 5). The prognostic nomograms was a predictive tool for estimating the 1-year DFS and OS of these patients. Each variable in the nomogram is assigned a point scale to represent its relative contribution to the DFS/OS, and the number of points for each variable is determined by drawing a line upward to the points axis. The total score is obtained by adding up the scores of all variables on the corresponding scale, which can be used intuitively to estimate the probability of survival. Additionally, the calibration curves were generated to suggest that the nomogram-predicted DFS/OS had good accordance with the actual DFS/OS (Figure 5). Besides, we captured ^18^F-FDG PET/CT images of two patients, as shown in the Figure 6.

## 4 Discussion

At present, there is an increasing amount of research investigating whether ^18^F-FDG PET/CT metabolic parameters can predict the prognosis of various types of cancer. Some studies have reported positive correlations between ^18^F-FDG PET/CT metabolic parameters and cancer prognosis. For instance, Dong X. et al. 2018 demonstrated that the PET/CT parameter SUV_max_ was closely related to the prognosis of myeloma patients (Dong et al., 2024). Similarly, Wakisaka R. et al. 2024 concluded that post-SUV_max_ could serve as an independent parameter for predicting poor survival in head and neck cancer (Wakisaka et al., 2024). However, other studies have reported contradictory findings, such as Collarino et al. (2024) found that in a large series of locally advanced cervical cancer patients treated with neoadjuvant chemoradiotherapy followed by surgery, PET radiomic features could not predict histopathologic tumor response and survival (Collarino et al., 2024). Additionally, Schröer-Günther M. et al. 2015 stated that there is no robust evidence for a patient-relevant benefit of PET and PET-CT in patients with esophageal carcinoma (Schröer-Günther et al., 2015).

Currently, most studies indicate that PET/CT parameters of primary tumors are closely related to the prognosis of breast cancer patients, although some studies have not reached a positive conclusion. In the study by Yue Y. et al. 2015 involving 79 TNBC patients, pre-treatment ^18^F-FDG PET/CT imaging had significant prognostic value in predicting the survival outcomes of TNBC patients (Yue et al., 2015). And Qu Y. H. et al. 2021 found that the metabolic parameters of ^18^F-FDG PET/CT in breast cancer can reflect the biological behavior of the tumor indirectly (Qu et al., 2021). Evangelista L. et al. 2023 conducted a systematic review by using three different databases and found that PET metrics were helpful for the prognosis stratification in patients with locally advanced or metastatic BC, however, no specific cut-off values for these variables were now available in this setting of patients (Evangelista et al., 2023). Meanwhile, in the study by Kim Y. I. et al. 2017, although all lymph node metabolic and volumetric parameters showed significant differences between patients with and without recurrence, the metabolic and volume parameters of primary breast tumors had no significant impact on prognosis (Kim et al., 2017).

Furthermore, an increasing number of studies are exploring the relationship between PET/CT imaging omics parameters, including metabolic parameters, and tumor prognosis. The meta-analysis of 975 patients with primary breast cancer by Pak K. et al. 2020 showed that patients with a high MTV or TLG from primary tumor have a higher risk of adverse events and patients with a high TLG from whole-body tumor have a higher risk of deaths (Pak et al., 2020). Najid S. et al. 2023 confirmed that high TMTV in breast cancer before treatment is associated with no pathologic complete response and recurrence (Najid et al., 2023). In the study by Seban R. D. et al. 2023, TNBC patients with high SUV_max_ and low TMTV were more likely to achieve pCR regardless of whether they received neoadjuvant therapy (Seban et al., 2023). In this study, we applied Kaplan-Meier survival and Cox regression analysis to explore the relationship between ^18^F-FDG PET/CT parameters and the prognosis of TNBC patients post-surgery. We found that SUV_mean_ was significantly correlated with DFS, but we did not find any other metabolic and volume parameters affecting the prognosis of these patients.

In the literature of the past few years, it was mentioned that fibrinogen level, D-dimer level, and the ratio of lymphocytes to monocytes are independent predictors of achieving pCR in patients with TNBC receiving neoadjuvant chemotherapy (Zhang et al., 2019). The ratio of neutrophils to lymphocytes, angiotensin-converting enzyme 2, total protein, blood urea nitrogen, cystatin C, potassium, phosphorus, platelet distribution width, activated partial thromboplastin time, thrombin time, and hepatitis B surface antibody are closely related to the prognosis of breast cancer (Tiainen et al., 2021; Nair et al., 2021; Li et al., 2021). However, none of the listed prognostic factors in blood serum are generally accepted in clinical practice (Bel’skaya and Sarf, 2022). We conducted statistical analysis on the relationship between biochemical indicators and the prognosis of TNBC patients post-surgery, and found that FBG, ALT, AFU and CK were independent predictors of DFS in these patients, while PALB and CK were independent predictors of OS. Additionally, we explored correlations between biochemical indicators and ^18^F-FDG PET/CT parameters. We found that PALB was correlated with all ^18^F-FDG PET/CT parameters; ALB was correlated with TLG, MTV and TMTV; FBG was correlated with SUV_max_, SUV_mean_ and SUV_peak_; and CK was correlated with SUV_peak_. The results indicated that ^18^F-FDG PET/CT parameters and biochemical indicators have potential utility in constituting a new prognostic model for TNBC patients post-surgery. FBG and CK were selected as two biochemical indicators that can independently predict the prognosis of TNBC patients post-surgery and are related to ^18^F-FDG PET/CT parameters.

At present, it is uncertain whether glucose levels are associated with the survival rate of any or all cancer patients. Despite the lack of scientific data, many cancer patients believe that glucose “feeds” cancer, which may affect disease outcomes (Boursi et al., 2016). In previous studies evaluating FBG and the prognosis of BC patients, the results were contradictory. In Minicozzi et al. 2013’s study, in ER+ PR+ female BC patients, after adjusting for other variables, patients with blood glucose levels >94 mg/dL had a higher mortality rate than those with blood glucose levels between 84.1 and 94.0 mg/dL, and demonstrated that high FBG and BMI are independently associated with an increased risk of mortality in BC (Minicozzi et al., 2013). In a cohort study on fasting blood glucose and non metastatic BC, Contiero P. et al. 2013 found that FBG and BMI have an independent impact on the prognosis of BC, and high FBG and obesity during the perioperative period indicate a deterioration in the short-term and long-term prognosis of BC patients (Contiero et al., 2013). Nevertheless, in the study by Wulaningsih W. et al. 2015, COX regression analysis found a lack of correlation between pre diagnostic blood glucose, triglycerides, or total cholesterol and BC mortality (Wulaningsih et al., 2015). Currently, there is no research which reports the correlation between FBG and the prognosis of TNBC patients. In our study, positive results were obtained in COX regression analysis indicating that FBG was a predictive factor for prognosis of TNBC patients post-surgery. This provides strong evidence for glucose levels to predict the prognosis of TNBC patients.

CK is a key enzyme in skeletal muscle contraction and relaxation energy metabolism, and is associated with mitochondrial oxidative phosphorylation, distributed in the myocardium, skeletal muscle, central nervous system, and smooth muscle (Shoji, 1995). In a case-control study accommodating 823 BC patients, there was a negative correlation between serum CK levels and tumor size or cancer stage (Pan et al., 2013). In our study, we confirmed that CK is an independent predictor of DFS in TNBC patients post-surgery. And the multivariate COX regression analysis indicated that CK was closely correlated with OS in these patients. In research on other types of cancer, CK is closely related to cancer prognosis. In bladder cancer, low preoperative CK levels independently predict worse survival results of bladder cancer after radical cystectomy (Li et al., 2024). In gastric cancer, low CK levels are an independent adverse prognostic factor for male patients (Yamazaki et al., 2022). In lung cancer, CK and LDH can serve as independent factors for predicting poor prognosis in adenocarcinoma patients (Liu et al., 2017). And Jiang Y. et al. 2021 confirmed that in non-small cell lung cancer treated with tyrosine kinase inhibitors, higher baseline CK levels and significant increase in CK after treatment were both associated with prolonged progression free survival (Jiang et al., 2021).

Firstly, as this study is a single center retrospective study with a small number of included cases and the follow-up results are not complete, which reduces the statistical validity and may lead to bias in the statistical results. Therefore, larger scale sample validation studies should be conducted in different institutions to further validate our research results. Secondly, the biochemical indicators included in this study are not extensive enough. If future research incorporates biochemical indicators such as blood routine, coagulation function, electrolytes, etc. on this basis, more indicators related to breast cancer prognosis and ^18^F-FDG PET/CT parameters may be screened out, providing a basis for biochemical indicators to predict prognosis of TNBC patients together with ^18^F-FDG PET/CT parameters, so as to bring more rigorous prediction methods to patients.

## 5 Conclusion

This study identifies SUV_mean_, FBG, AFU, and CK are predictive factors for DFS in TNBC patients post-surgery, and PALB and CK are predictive factors for OS, so we could pay more attention to these indicators in clinical practice. The integration of ^18^F-FDG PET/CT parameters and biochemical indicators enhances the prognostic evaluation of TNBC patients, potentially improving clinical outcomes.

## Data Availability

The original contributions presented in the study are included in the article/Supplementary Material, further inquiries can be directed to the corresponding authors.

## Supplementary material

SUPPLEMENTARY FIGURE S1 The negative results of ^18^F-FDG PET/CT parameters in the Kaplan Meier survival curves of DFS and OS in TNBC patients post-surgery. **(A)** MTV with DFS; **(B)** SUV_max_ with DFS; **(C)** TLG with DFS; **(D)** TMTV with DFS; **(E)** MTV with OS; **(F)** SUV_max_ with OS; **(G)** SUV_mean_ with OS; **(H)** SUV_peak_ with OS; **(I)** TLG with OS; **(J)** TMTV with OS.

SUPPLEMENTARY FIGURE S2 The negative results of clinical and pathological features in the Kaplan Meier survival curves of DFS in TNBC patients post-surgery. **(A)** Age at diagnosis with DFS; **(B)** CK5/6 with DFS; **(C)** EGFR with DFS; **(D)** Ki67 with DFS; **(E)** P53 with DFS; **(F)** maximum transverse diameter of tumor with DFS; **(G)** maximum longitudinal diameter of tumor with DFS; **(H)** Axillary lymph node metastasis with DFS; **(I)** diabetes mellitus with DFS; **(J)** Histological grade with DFS; **(K)** menopause with DFS.

SUPPLEMENTARY FIGURE S3 The negative results of clinical and pathological features in the Kaplan Meier survival curves of OS in TNBC patients post-surgery. **(A)** Age at diagnosis with OS; **(B)** CK5/6 with OS; **(C)** EGFR with OS; **(D)** Ki67 with OS; **(E)** P53 with OS; **(F)** maximum transverse diameter of tumor with OS; **(G)** maximum longitudinal diameter of tumor with OS; **(H)** Axillary lymph node metastasis with OS; **(I)** chemotherapy response with OS; **(J)** diabetes mellitus with OS; **(K)** Histological grade with OS; **(L)** menopause with OS.

SUPPLEMENTARY FIGURE S4 The negative results of Biochemical indicators in the Kaplan Meier survival curves of DFS in TNBC patients post-surgery.

SUPPLEMENTARY FIGURE S5 The negative results of Biochemical indicators in the Kaplan Meier survival curves of OS in TNBC patients post-surgery.

SUPPLEMENTARY TABLE S1 Univariate Cox regression analysis of ^18^F-FDG PET/CT parameters with DFS and OS in TNBC patients post-surgery.

SUPPLEMENTARY TABLE S2 The negative results of univariate Cox regression analysis of Biochemical indicators with DFS in TNBC patients post-surgery.

SUPPLEMENTARY TABLE S3 The negative results of univariate Cox regression analysis of biochemical indicators with OS in TNBC patients post-surgery.

SUPPLEMENTARY TABLE S4
*P* value of the correlation between 18F-FDG PET/CT parameters and biochemical indicators.

## References

[B1] Alam M. S. Sultana A. Wang G. Haque Mollah M. N. (2022). Gene expression profile analysis to discover molecular signatures for early diagnosis and therapies of triple-negative breast cancer. Front. Mol. Biosci. 9, 1049741. 10.3389/fmolb.2022.1049741 36567949 PMC9768339

[B2] Bel’skaya L. V. Sarf E. A. (2022). Prognostic value of salivary biochemical indicators in primary resectable breast cancer. Metabolites 12 (6), 552. 10.3390/metabo12060552 35736486 PMC9227854

[B3] Bergin A. Loi S. (2019). Triple-negative breast cancer: recent treatment advances. F1000Res 8, F1000 Faculty Rev-1342. 10.12688/f1000research.18888.1 PMC6681627 31448088

[B4] Boellaard R. Delgado-Bolton R. Oyen W. J. Giammarile F. Tatsch K. Eschner W. (2015). FDG PET/CT: EANM procedure guidelines for tumour imaging: version 2.0. Eur. J. Nucl. Med. Mol. Imaging 42 (2), 328–354. 10.1007/s00259-014-2961-x 25452219 PMC4315529

[B5] Bouron C. Mathie C. Seegers V. Morel O. Jézéquel P. Lasla H. (2022). Prognostic value of metabolic, volumetric and textural parameters of baseline [(18)F]FDG PET/CT in early triple-negative breast cancer. Cancers (Basel) 14 (3), 637. 10.3390/cancers14030637 35158904 PMC8833829

[B6] Boursi B. Giantonio B. J. Lewis J. D. Haynes K. Mamtani R. Yang Y. X. (2016). Serum glucose and hemoglobin A1C levels at cancer diagnosis and disease outcome. Eur. J. Cancer 59, 90–98. 10.1016/j.ejca.2016.02.018 27017290 PMC4851868

[B7] Cardoso F. Kyriakides S. Ohno S. Penault-Llorca F. Poortmans P. Rubio I. T. (2019). Early breast cancer: ESMO Clinical Practice Guidelines for diagnosis, treatment and follow-up. Ann. Oncol. 30 (8), 1194–1220. 10.1093/annonc/mdz173 31161190

[B8] Collarino A. Feudo V. Pasciuto T. Florit A. Pfaehler E. de Summa M. (2024). Is PET radiomics useful to predict pathologic tumor response and prognosis in locally advanced cervical cancer. J. Nucl. Med. 123, 267044. 10.2967/jnumed.123.267044 38548352

[B9] Contiero P. Berrino F. Tagliabue G. Mastroianni A. Di Mauro M. G. Fabiano S. (2013). Fasting blood glucose and long-term prognosis of non-metastatic breast cancer: a cohort study. Breast Cancer Res. Treat. 138 (3), 951–959. 10.1007/s10549-013-2519-9 23568483 PMC3664213

[B10] de Mooij C. M. Ploumen R. Nelemans P. J. Mottaghy F. M. Smidt M. L. van Nijnatten T. (2023). The influence of receptor expression and clinical subtypes on baseline [18F]FDG uptake in breast cancer: systematic review and meta-analysis. EJNMMI Res. 13 (1), 5. 10.1186/s13550-023-00953-y 36689007 PMC9871105

[B11] Dong X. Wang R. Ying X. Xu J. Yan J. Xu P. (2024). Construction and validation of an (18)F-FDG-PET/CT-based prognostic model to predict progression-free survival in newly diagnosed multiple myeloma patients. Hematology 29 (1), 2329029. 10.1080/16078454.2024.2329029 38488443

[B12] Evangelista L. Urso L. Caracciolo M. Stracuzzi F. Panareo S. Cistaro A. (2023). FDG PET/CT volume-based quantitative data and survival analysis in breast cancer patients: a systematic review of the literature. Curr. Med. Imaging 19 (8), 807–816. 10.2174/1573405618666220329094423 35352652

[B13] Fowler J. S. Ido T. (2002). Initial and subsequent approach for the synthesis of 18FDG. Semin. Nucl. Med. 32 (1), 6–12. 10.1053/snuc.2002.29270 11839070

[B14] Humbert O. Berriolo-Riedinger A. Riedinger J. M. Coudert B. Arnould L. Cochet A. (2012). Changes in 18F-FDG tumor metabolism after a first course of neoadjuvant chemotherapy in breast cancer: influence of tumor subtypes. Ann. Oncol. 23 (10), 2572–2577. 10.1093/annonc/mds071 22499859

[B15] Jiang Y. Su Z. Lin Y. Xiong Y. Li C. Li J. (2021). Prognostic and predictive impact of creatine kinase level in non-small cell lung cancer treated with tyrosine kinase inhibitors. Transl. Lung Cancer Res. 10 (9), 3771–3781. 10.21037/tlcr-21-600 34733627 PMC8512461

[B16] Kim Y. I. Kim Y. J. Paeng J. C. Cheon G. J. Lee D. S. Chung J. K. (2017). Prediction of breast cancer recurrence using lymph node metabolic and volumetric parameters from (18)F-FDG PET/CT in operable triple-negative breast cancer. Eur. J. Nucl. Med. Mol. Imaging 44 (11), 1787–1795. 10.1007/s00259-017-3748-7 28616695

[B17] Koo H. R. Park J. S. Kang K. W. Cho N. Chang J. M. Bae M. S. (2014). 18F-FDG uptake in breast cancer correlates with immunohistochemically defined subtypes. Eur. Radiol. 24 (3), 610–618. 10.1007/s00330-013-3037-1 24097303

[B18] Li Y. Xu H. Lin T. Zhang J. Ai J. Zhang S. (2024). Preoperative low plasma creatine kinase levels predict worse survival outcomes in bladder cancer after radical cystectomy. Int. Urol. Nephrol. 56, 2215–2225. 10.1007/s11255-024-03957-2 38315281

[B19] Li Y. Zhang J. Wang B. Zhang H. He J. Wang K. (2021). A nomogram based on clinicopathological features and serological indicators predicting breast pathologic complete response of neoadjuvant chemotherapy in breast cancer. Sci. Rep. 11 (1), 11348. 10.1038/s41598-021-91049-x 34059778 PMC8167133

[B20] Liu L. He Y. Ge G. Li L. Zhou P. Zhu Y. (2017). Lactate dehydrogenase and creatine kinase as poor prognostic factors in lung cancer: a retrospective observational study. PLoS One 12 (8), e0182168. 10.1371/journal.pone.0182168 28767733 PMC5540491

[B21] Minicozzi P. Berrino F. Sebastiani F. Falcini F. Vattiato R. Cioccoloni F. (2013). High fasting blood glucose and obesity significantly and independently increase risk of breast cancer death in hormone receptor-positive disease. Eur. J. Cancer 49 (18), 3881–3888. 10.1016/j.ejca.2013.08.004 24011933

[B22] Nair M. G. Prabhu J. S. Ts S. (2021). High expression of ACE2 in HER2 subtype of breast cancer is a marker of poor prognosis. Cancer Treat. Res. Commun. 27, 100321. 10.1016/j.ctarc.2021.100321 33517235 PMC7825889

[B23] Najid S. Seban R. D. Champion L. De Moura A. Sebbag C. Salaün H. (2023). Clinical utility of pre-therapeutic [18F]FDG PET/CT imaging for predicting outcomes in breast cancer. J. Clin. Med. 12 (17), 5487. 10.3390/jcm12175487 37685551 PMC10488013

[B24] Nguyen P. Bhatt M. Bashirzadeh F. Hundloe J. Ware R. Fielding D. (2015). Comparison of objective criteria and expert visual interpretation to classify benign and malignant hilar and mediastinal nodes on 18-F FDG PET/CT. Respirology 20 (1), 129–137. 10.1111/resp.12409 25263085

[B25] Pak K. Seok J. W. Kim H. Y. Nguyen T. L. Kim S. J. Kim K. (2020). Prognostic value of metabolic tumor volume and total lesion glycolysis in breast cancer: a meta-analysis. Nucl. Med. Commun. 41 (8), 824–829. 10.1097/MNM.0000000000001227 32516244

[B26] Pan H. Xia K. Zhou W. Xue J. Liang X. Cheng L. (2013). Low serum creatine kinase levels in breast cancer patients: a case-control study. PLoS One 8 (4), e62112. 10.1371/journal.pone.0062112 23614022 PMC3626709

[B27] Paydary K. Seraj S. M. Zadeh M. Z. Emamzadehfard S. Shamchi S. P. Gholami S. (2019). The evolving role of FDG-PET/CT in the diagnosis, staging, and treatment of breast cancer. Mol. Imaging Biol. 21 (1), 1–10. 10.1007/s11307-018-1181-3 29516387

[B28] Qu Y. H. Long N. Ran C. Sun J. (2021). The correlation of (18)F-FDG PET/CT metabolic parameters, clinicopathological factors, and prognosis in breast cancer. Clin. Transl. Oncol. 23 (3), 620–627. 10.1007/s12094-020-02457-w 32683540

[B29] Schöder H. Moskowitz C. (2016). Metabolic tumor volume in lymphoma: hype or hope. J. Clin. Oncol. 34 (30), 3591–3594. 10.1200/JCO.2016.69.3747 27601547 PMC5547014

[B30] Schröer-Günther M. Scheibler F. Wolff R. Westwood M. Baumert B. Lange S. (2015). The role of PET and PET-CT scanning in assessing response to neoadjuvant therapy in esophageal carcinoma. Dtsch. Arztebl Int. 112 (33-34), 545–552. 10.3238/arztebl.2015.0545 26356551 PMC4570959

[B31] Seban R. D. Arnaud E. Loirat D. Cabel L. Cottu P. Djerroudi L. (2023). [18F]FDG PET/CT for predicting triple-negative breast cancer outcomes after neoadjuvant chemotherapy with or without pembrolizumab. Eur. J. Nucl. Med. Mol. Imaging 50 (13), 4024–4035. 10.1007/s00259-023-06394-y 37606858

[B32] Shoji S. (1995). Creatine kinase (CK). Nihon Rinsho 53 (5), 1136–1140. 7602768

[B33] Sung H. Ferlay J. Siegel R. L. Laversanne M. Soerjomataram I. Jemal A. (2021). Global cancer statistics 2020: GLOBOCAN estimates of incidence and mortality worldwide for 36 cancers in 185 countries. CA Cancer J. Clin. 71 (3), 209–249. 10.3322/caac.21660 33538338

[B34] Tiainen S. Rilla K. Hämäläinen K. Oikari S. Auvinen P. (2021). The prognostic and predictive role of the neutrophil-to-lymphocyte ratio and the monocyte-to-lymphocyte ratio in early breast cancer, especially in the HER2+ subtype. Breast Cancer Res. Treat. 185 (1), 63–72. 10.1007/s10549-020-05925-7 32948994 PMC7500503

[B35] Wakisaka R. Kumai T. Komatsuda H. Yamaki H. Kono M. Sato R. (2024). Prognostic value of the (18)F-FDG PET/CT and haematological parameters in head and neck cancer. Clin. Otolaryngol. 10.1111/coa.14195 38950901

[B36] Wolff A. C. Hammond M. E. Hicks D. G. Dowsett M. McShane L. M. Allison K. H. (2013). Recommendations for human epidermal growth factor receptor 2 testing in breast cancer: American Society of Clinical Oncology/College of American Pathologists clinical practice guideline update. J. Clin. Oncol. 31 (31), 3997–4013. 10.1200/JCO.2013.50.9984 24101045

[B37] Wulaningsih W. Vahdaninia M. Rowley M. Holmberg L. Garmo H. Malmstrom H. (2015). Prediagnostic serum glucose and lipids in relation to survival in breast cancer patients: a competing risk analysis. BMC Cancer 15, 913. 10.1186/s12885-015-1928-z 26577580 PMC4650114

[B38] Xu P. Wang Y. (2020). Application of (18)F-FDG PET/CT in evaluation of curative effect and prognosis for small cell lung cancer. Zhong Nan Da Xue Xue Bao Yi Xue Ban. 45 (10), 1255–1260. 10.11817/j.issn.1672-7347.2020.190151 33268589

[B39] Yamazaki N. Oshima Y. Shiratori F. Nanami T. Suzuki T. Yajima S. (2022). Prognostic significance of preoperative low serum creatine kinase levels in gastric cancer. Surg. Today 52 (11), 1551–1559. 10.1007/s00595-022-02505-8 35478264

[B40] Yan Z. Zhong Z. Shi C. Feng M. Feng X. Liu T. (2024). The prognostic marker KRT81 is involved in suppressing CD8 + T cells and predicts immunotherapy response for triple-negative breast cancer. Cancer Biol. Ther. 25 (1), 2355705. 10.1080/15384047.2024.2355705 38778753 PMC11123506

[B41] Yue Y. Cui X. Bose S. Audeh W. Zhang X. Fraass B. (2015). Stratifying triple-negative breast cancer prognosis using 18F-FDG-PET/CT imaging. Breast Cancer Res. Treat. 153 (3), 607–616. 10.1007/s10549-015-3558-1 26346756 PMC4589560

[B42] Zhang F. Huang M. Zhou H. Chen K. Jin J. Wu Y. (2019). A nomogram to predict the pathologic complete response of neoadjuvant chemotherapy in triple-negative breast cancer based on simple laboratory indicators. Ann. Surg. Oncol. 26 (12), 3912–3919. 10.1245/s10434-019-07655-7 31359285

