# Supplementary material for: Evaluation of prognostic risk factors of triple-negative breast cancer with 18F-FDG PET/CT parameters, clinical pathological features and biochemical indicators
Source: Front Cell Dev Biol. 2024 Sep 4;12:1421981. doi: 10.3389/fcell.2024.1421981 (PMC11408346; doi:10.3389/fcell.2024.1421981)
Supplement: Supplementary file 1 [file Table1.DOCX]

Table S1 Univariate Cox regression analysis of ^18^F-FDG PET/CT parameters with DFS and OS in TNBC patients who underwent surgery.

| ^18^F-FDG PET/CT parameters | | DFS | | OS | |
| --- | --- | --- | --- | --- | --- |
|  |  | HR(95%CI) | P.Value | HR(95%CI) | P.Value |
| SUVmax | 1.031(0.965-1.102) | | 0.359 | 0.973(0.888-1.066) | 0.56 |
| SUVmean | 0.923(0.699-1.218) | | 0.571 | 0.835(0.582-1.199) | 0.329 |
| SUVpeak | 1.04(0.963-1.123) | | 0.316 | 0.96(0.855-1.077) | 0.485 |
| TLG | 1(0.999-1) | | 0.823 | 0.999(0.995-1.002) | 0.433 |
| MTV | 0.999(0.994-1.004) | | 0.72 | 0.992(0.97-1.014) | 0.454 |
| TMTV | 1(0.996-1.003) | | 0.914 | 0.994(0.979-1.009) | 0.431 |
