# Supplementary figures and images for: Evaluation of prognostic risk factors of triple-negative breast cancer with 18F-FDG PET/CT parameters, clinical pathological features and biochemical indicators
Source: Front Cell Dev Biol. 2024 Sep 4;12:1421981. doi: 10.3389/fcell.2024.1421981 (PMC11408346; doi:10.3389/fcell.2024.1421981)

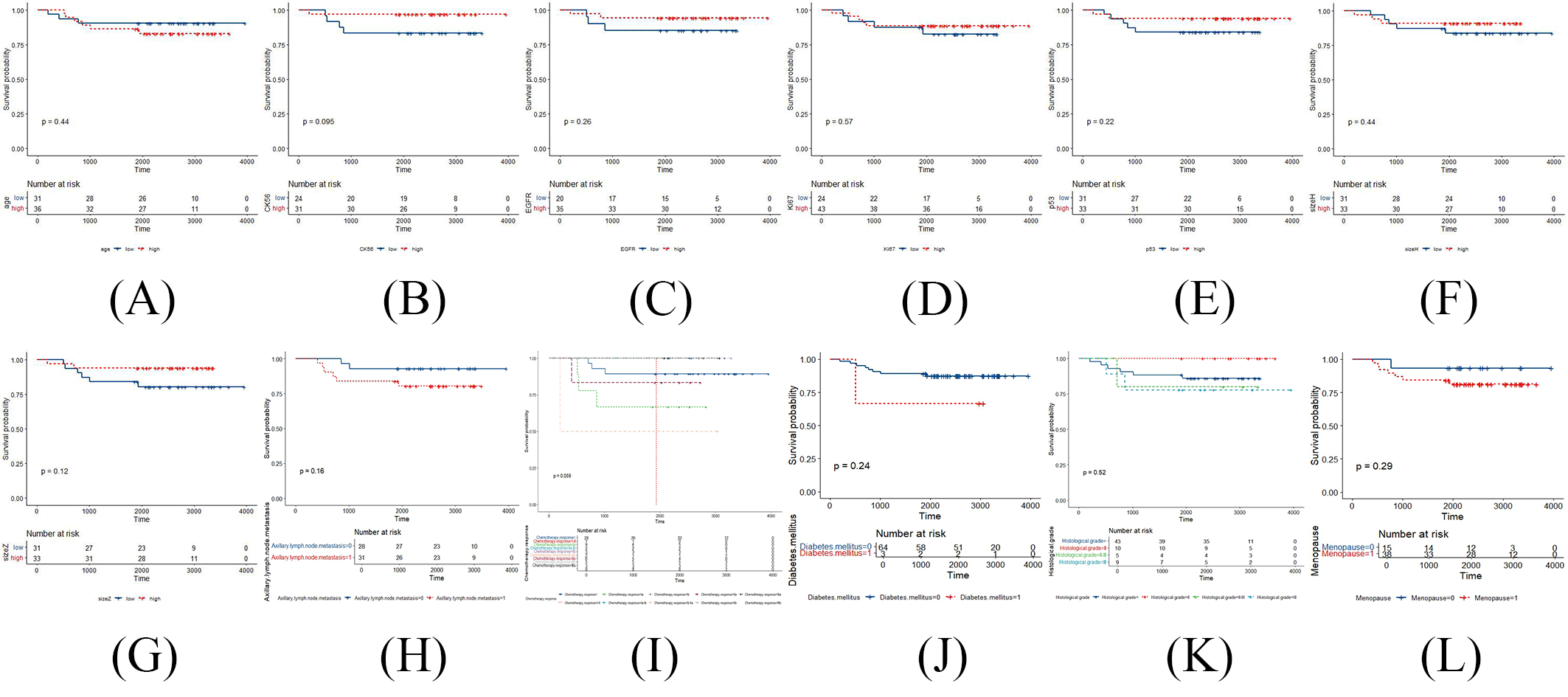

Supplement: Supplementary file 3 [file Image3.TIF]

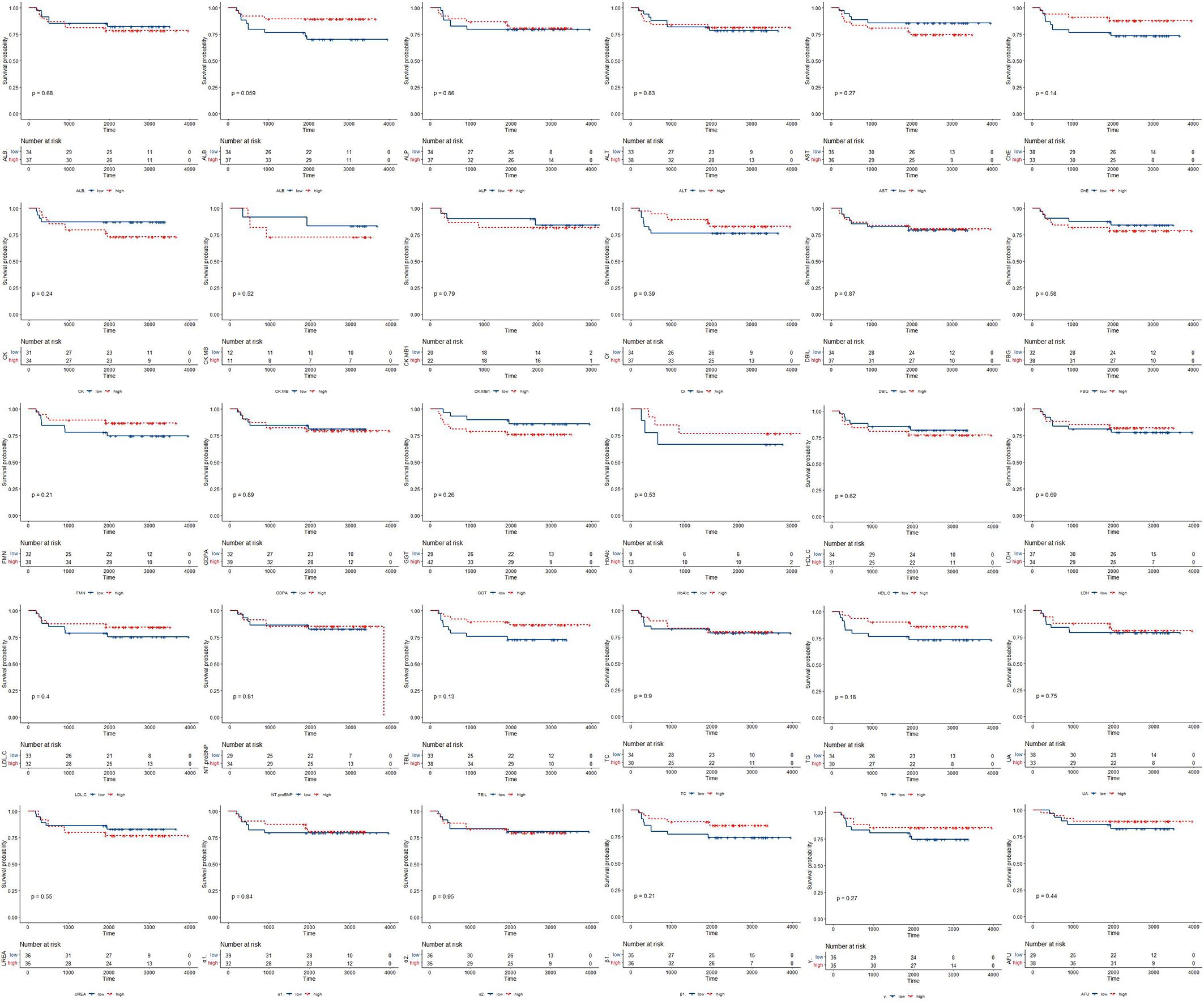

Supplement: Supplementary file 5 [file Image4.TIF]

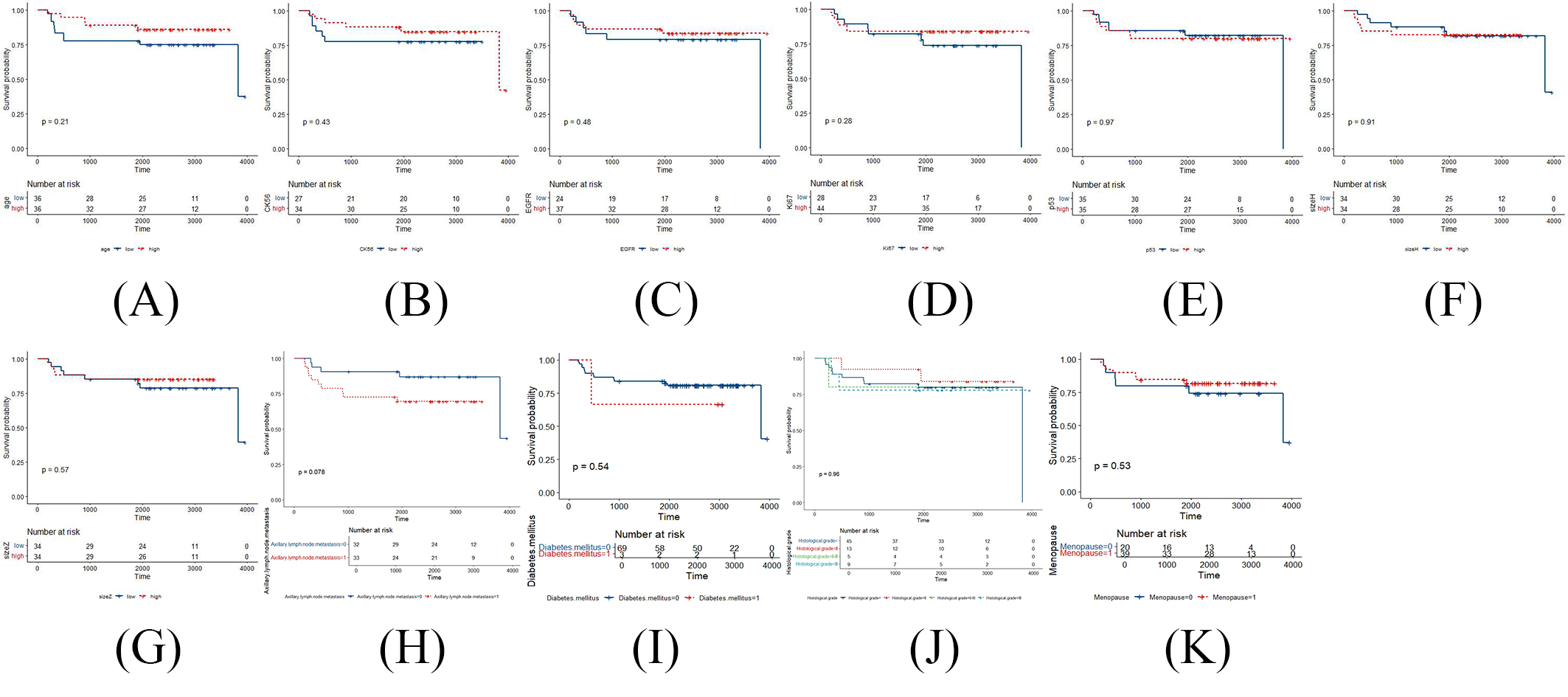

Supplement: Supplementary file 6 [file Image2.TIF]

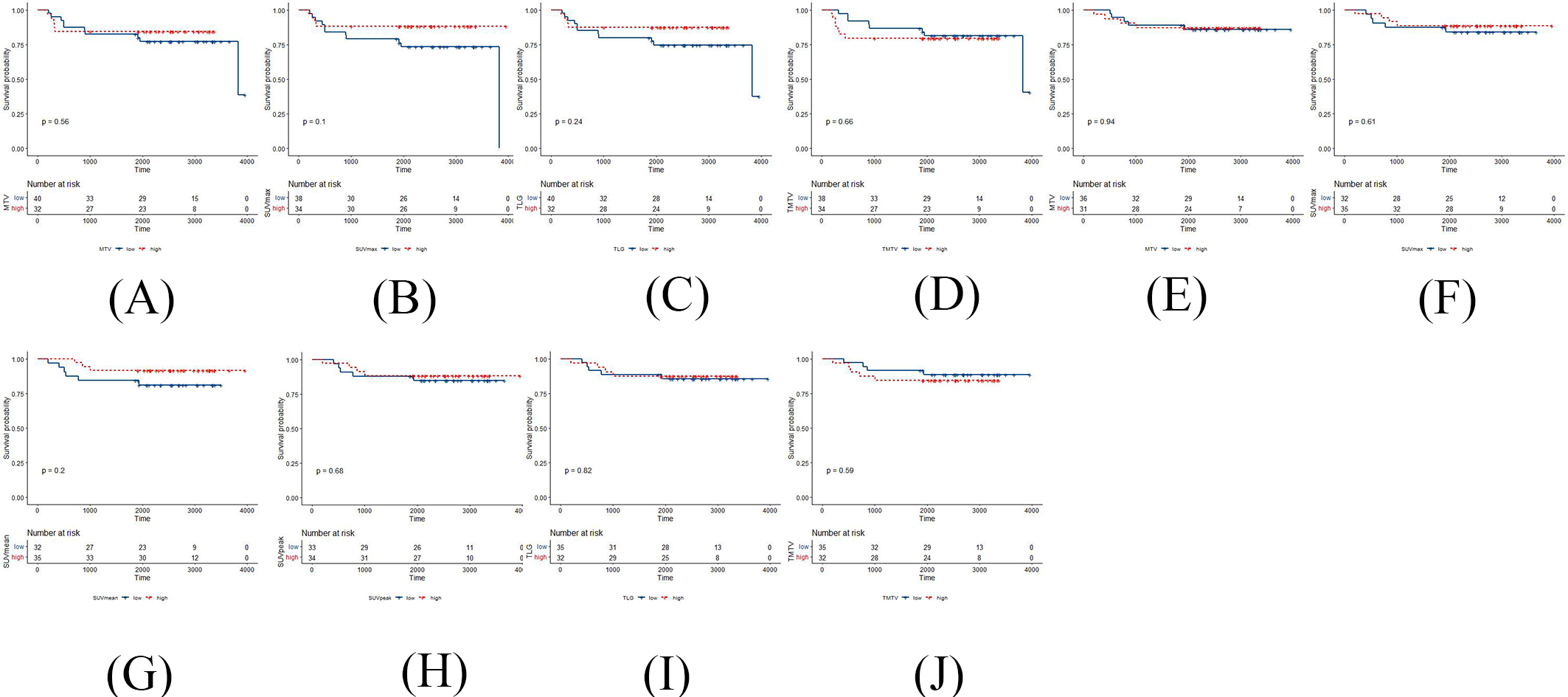

Supplement: Supplementary file 7 [file Image1.TIF]

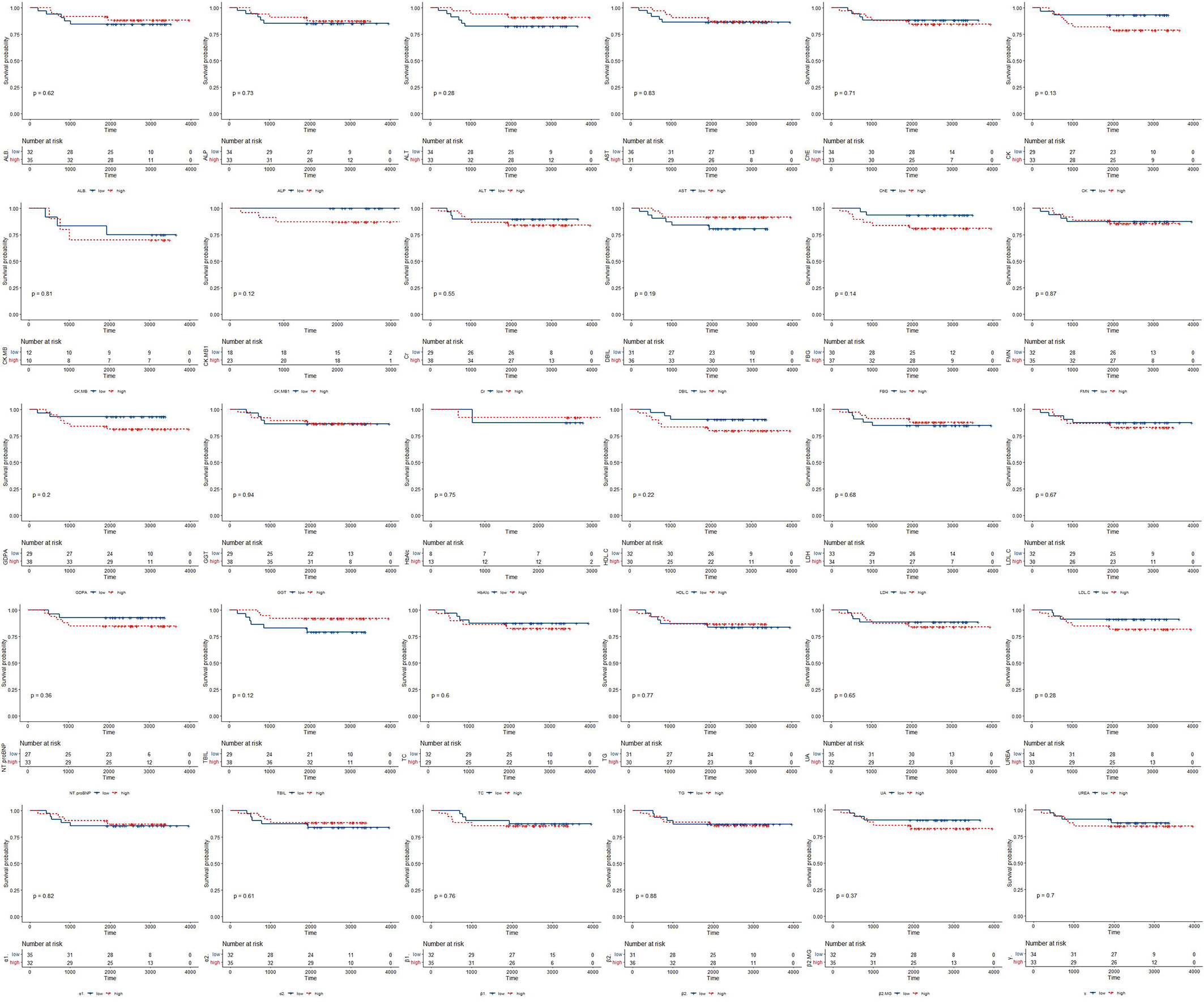

Supplement: Supplementary file 9 [file Image5.TIF]
